# A Cystic Variant of Acinar Cell Carcinoma of the Pancreas with Repeated Bleeding from a Pseudoaneurysm: A Case Report

**DOI:** 10.70352/scrj.cr.25-0273

**Published:** 2025-10-21

**Authors:** Kazue Morishima, Kazuhiro Endo, Hideki Sasanuma, Yasunaru Sakuma, Noriyoshi Fukushima, Hironori Yamaguchi, Naohiro Sata

**Affiliations:** 1 Department of Surgery, Division of Gastroenterological, General and Transplant Surgery, Jichi Medical University, Shimotsuke, Tochigi, Japan; 2 Department of Pathology, Jichi Medical University, Shimotsuke, Tochigi, Japan

**Keywords:** acinar cell carcinoma, pseudoaneurysm, cystic formation, pancreatic pseudocyst

## Abstract

**INTRODUCTION:**

Acinar cell carcinomas are rare pancreatic neoplasms, accounting for approximately 1% of all exocrine pancreatic tumors. We describe a case of a cystic variant with intracystic hemorrhaging that was difficult to differentiate from a pseudocyst due to its morphology.

**CASE PRESENTATION:**

A 54-year-old man was admitted with severe left upper quadrant abdominal pain. Imaging studies showed a 7.0-cm internal heterogeneous cystic lesion with a splenic artery pseudoaneurysm near the lesion. Transarterial embolization of the splenic artery was performed, but rebleeding occurred 1 month later. Distal pancreatectomy with partial resection of the stomach revealed internal nodular lesions on the resected specimen. Microscopically, the cystic mass was composed of neoplastic tissue with papillary and tubular structures. The tumor was diagnosed as acinar cell carcinoma since immunohistochemical examination showed tumor cells positive for BCL10, lipase, and trypsin. The patient experienced local recurrence 6 months postoperatively, received chemotherapy with gemcitabine followed by S-1, underwent a 2nd resection at 18 months, and has remained recurrence-free for 15 years.

**CONCLUSIONS:**

Acinar cell carcinoma rarely presents with a cystic structure and may be accompanied by a pseudoaneurysm, which can complicate differentiation from a pancreatic pseudocyst, highlighting the importance of careful differential diagnosis for appropriate treatment.

## Abbreviations

EUS
: endoscopic ultrasound
TAE
: trans-arterial embolization

## INTRODUCTION

Acinar cell carcinomas are rare neoplasms of the pancreas, accounting for approximately 1% of all exocrine pancreatic tumors,^1)^ occurring predominantly in late adulthood and more frequently in males. They are typically large when detected and appear well marginated, with a thin, enhancing capsule. Enhanced CT scans show a hypervascular lesion usually having central hypodensity with necrosis and calcification. Cystic formation is a very rare variant and is mainly reported as acinar cell cystadenocarcinomas, characterized by large, multilocular cystic masses replacing the body and tail of the pancreas.^2)^ Microscopically, they have multiple cysts and tubules lined by a single layer of cuboidal or columnar cells with basal nuclei and cytoplasmic characteristics similar to solid tumors. Intraductal growth has been reported in the literature in individual case reports.^3,4)^ Basturk et al.^5)^ reported 7 acinar cell carcinoma patients with intraductal, papillary, or papillocystic growth patterns that can mimic intraductal papillary mucinous neoplasms. All cases had cystic areas, but no mucin was identified.

Most acinar cell carcinomas are asymptomatic. The most common clinical presentations include weight loss, abdominal pain, nausea, vomiting, and elevated serum lipase levels.^6)^ Elevated lipase levels can lead to systemic manifestations such as subcutaneous fat necrosis and polyarthritis, and a rare case of recurrent pancreatitis secondary to pancreatic acinar cell carcinoma has been reported.^7)^ Spontaneous rupture of the carcinoma presenting as an acute abdomen has also been reported.^8)^

Here, we describe a patient with a cystic variant of acinar cell carcinoma in which a pseudoaneurysm of the splenic artery ruptured into the tumor, resulting in repeated episodes of intratumoral hemorrhage. To our knowledge, this is the 1st report of such a case.

## CASE PRESENTATION

A 54-year-old man presented to an outside facility with left upper quadrant abdominal pain, and a 3.0-cm cystic mass was found in the pancreatic tail. Following a diagnosis of pancreatic pseudocyst, 1 month later he again developed left upper quadrant abdominal pain, and the cystic mass had enlarged to 6.0 cm with associated inflammatory changes. He was admitted to our institution with severe left upper quadrant abdominal pain the following month.

**Fig. 1:**
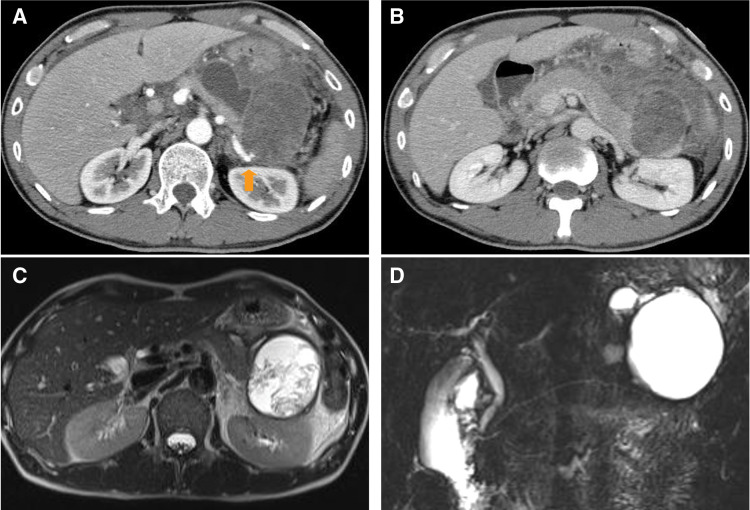
Imaging studies. (**A**) Contrast-enhanced CT scan showing a 7-cm heterogeneous cystic lesion with a pseudoaneurysm of the splenic artery in early-phase images (arrow). (**B**) Fluid collection with inflammatory changes was seen in late-phase images. (**C**) T2-weighted MRI showed a heterogeneous cystic lesion. (**D**) The main pancreatic duct was not dilated.

**Fig. 2:**
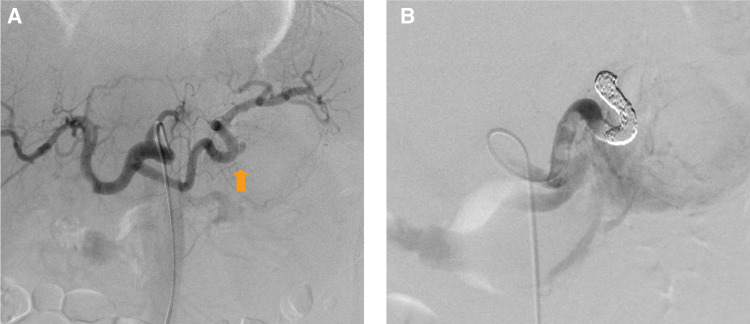
Celiac angiography. (**A**) A pseudoaneurysm of the splenic artery was observed (arrow). (**B**) Transcatheter arterial embolization was performed.

There was no history of acute or chronic pancreatitis. Laboratory data included: white blood cell count, 7.9 × 10^9^/L, hemoglobin 13.9 g/dL, C-reactive protein 6.5 mg/dL, serum amylase 58 mU/mL, lipase 107 U/L, carcinoembryonic antigen (CEA) 0.5 ng/mL, and carbohydrate antigen 19-9 (CA19-9) 6 U/mL. Abdominal CT scan showed a 7.0-cm heterogeneous cystic mass in the tail of the pancreas (**Fig. 1A** and **1B**). A pseudoaneurysm of the splenic artery was also seen near the cystic mass, with intracystic bleeding. Fluid collection and inflammatory changes were seen around the pancreas. The pancreatic duct was not dilated. The patient was diagnosed with acute pancreatitis and a pseudocyst with intracystic bleeding. Celiac angiography revealed that the pseudoaneurysm arose from the splenic artery, so TAE was successfully performed (**Fig. 2A** and **2B**).

After embolization, MRI showed a heterogeneous cystic lesion without an obvious solid component (**Fig. 1C** and **1D**). The pancreatic parenchyma and main pancreatic duct appeared normal, and the fluid collection had resolved. One month after TAE, the patient again developed abdominal pain. An abdominal CT scan showed hemorrhage within the cystic mass, and surgery was planned.

**Fig. 3:**
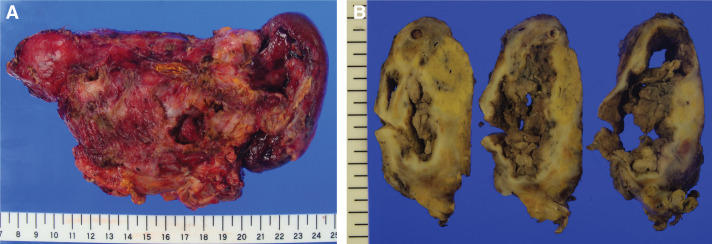
Resected specimen. (**A**) A 7.0-cm cystic mass was located in the tail of the pancreas. (**B**) The cystic tumor contained serous effusion and clots, and nodules were found.

During the procedure, a cystic lesion was found in the pancreatic tail, with thick fibrotic tissue of the cyst wall adhering to the posterior wall of the stomach. The pancreatic head and body appeared normal. Distal pancreatectomy and splenectomy with partial resection of the gastric wall were performed. Macroscopically, the cystic lesion, measuring 7 × 6 × 5 cm, contained serous fluid, clots, and some nodules without mucin (**Fig. 3A** and **3B**). The main pancreatic duct was obstructed, but communication with the cystic lesion was not found. The splenic artery was located near the cystic lesion.

**Fig. 4:**
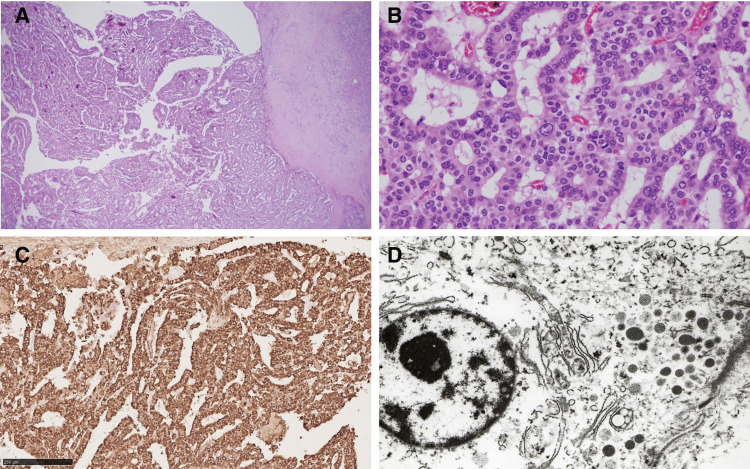
Histological and ultrastructural findings. (**A**) The nodules were composed of tumor tissue showing papillary and tubular structures (H&E stain, ×40). (**B**) Dysplastic cells had basophilic cytoplasm and round nuclei with prominent nucleoli (H&E stain, ×200). (**C**) The papillary tumor showed diffuse positivity for BCL10 (BCL10, scale bar 250 μm). (**D**) Electron microscopy confirmed the presence of 500-nm zymogen granules. H&E, hematoxylin and eosin

Microscopically, the cyst wall was composed of thick fibrotic tissue (**Fig. 4A** and **4B**). The nodules were composed of tumor tissue showing papillary and tubular structures. Dysplastic cells showed basophilic or pale eosinophilic cytoplasm and round nuclei. The epithelium of the pancreatic duct partially covered the lesion. The tumor had infiltrated the cyst wall and exhibited invasion into the splenic vein, as well as into the serosa and retroperitoneum. No lymph node metastasis was observed, and the pancreatic resection margin was negative. Although no tumor exposure at the dissection surface was identified, part of the thickened fibrotic tissue covering the tumor was disrupted. On the caudal side of the tumor, the embolized splenic artery was found in close contact with the tumor. Immunohistochemical staining revealed diffuse positivity for BCL10 and CPA1 (**Fig. 4C**). Trypsin, lipase, and synaptophysin also showed focal positivity, whereas chromogranin A was negative. Electron microscopy confirmed the presence of 500 nm zymogen granules (**Fig. 4D**). These findings were consistent with the diagnosis of acinar cell carcinoma.

There were no postoperative complications, but 6 months after resection, local recurrence was found near the superior pole of the left kidney. Gemcitabine was administered biweekly for a total of 6 cycles over 3 months. However, the tumor increased in size from 2.3 to 3.8 cm on CT imaging. As 2nd-line chemotherapy, S-1 was administered orally for 2 cycles over the next 3 months. Despite treatment, the tumor size further increased to 5.2 cm. Notably, the tumor markers CEA and CA19-9 remained within normal limits throughout the course, whereas serum trypsin levels increased from 1200 ng/mL at the initiation of S-1 to 7400 ng/mL after 4 months. No new lesions were detected during this period, and surgical resection of the recurrent tumor with left nephrectomy and partial resection of the gastric wall and left diaphragm was performed 18 months after the initial surgery.

Microscopically, the tumor was encapsulated by fibrous tissue and consisted of atypical cuboidal cells with pale eosinophilic, lipid-rich cytoplasm that proliferated in a glandular pattern. The histological features were consistent with those observed in the previously resected specimen, supporting the diagnosis of recurrent acinar cell carcinoma. No tumor cell exposure was observed at the dissection margin. The patient did not receive postoperative adjuvant chemotherapy, and no tumor relapse has been observed on follow-up 15 years after the 1st resection.

## DISCUSSION

To the best of our knowledge, this is the 14th reported case of cystic acinar cell carcinoma,^2,7,9,10)^ and the 1st case of a hemorrhagic pancreatic neoplasm due to a pseudoaneurysm associated with acinar cell carcinoma. Previously reported cases predominantly occurred in patients around 60 years of age (10 males and 4 females). Tumor location was the pancreatic head in 3 cases, the body or tail in 10 cases, and unknown in 1 case. Tumor size ranged from 3.0 to 39 cm, with a median size of 17 cm. Abdominal pain was reported in 7 cases. EUS is an excellent modality for visualizing the pancreas, and Aqel et al.^7)^ reported a case in which a solid component was identified by EUS.

This case involved a middle-aged man with a cystic lesion in the pancreatic body and tail, showing marked wall thickening consistent with previously reported features of cystic acinar cell carcinoma. Preoperative CT demonstrated a cystic lesion with internal heterogeneity, but no evident solid component was identified. A pseudocyst was initially suspected, but EUS was not performed. In retrospect, contrast-enhanced EUS might have revealed a solid component and facilitated a more accurate preoperative diagnosis.

Historically, 80%–90% of pancreatic cystic lesions have been considered to be pseudocysts, while the remaining 10%–20% are true cysts. Many of these true cysts are neoplastic and potentially malignant or premalignant.^11)^ It is important to distinguish between pancreatic pseudocysts and neoplastic cysts, especially potentially malignant intraductal papillary mucinous neoplasms, mucinous cystic neoplasms, solid pseudopapillary neoplasms, and cystic neuroendocrine neoplasms. Parenchymal calcifications and ductal changes consistent with chronic pancreatitis on CT, MRI, or EUS increase the likelihood of the cyst being a pseudocyst. On the other hand, calcification in the cyst wall and an otherwise normal pancreatic parenchyma suggest a neoplastic cyst. Solid components, mural excrescences, or septations within the cysts may be associated with neoplastic, including malignant, cystic lesions.^6)^

It is also important to differentiate between the cystic variant of acinar cell carcinoma and other cystic tumors of the pancreas. Pathological confirmation of zymogen granules or immunohistochemical staining for acinar enzymes is useful in differentiating these lesions from other pancreatic intraductal neoplasms. Microscopically, the lesion in the present case showed papillary and tubular structures with pancreatic epithelium partially surrounding the neoplasm, which suggests an intraductal neoplasm. Immunostaining was positive for pancreatic exocrine markers consistent with acinar cell carcinoma. Electron microscopy revealed a small number of cells with zymogen granules in the cytoplasm. The existing pancreatic ductal epithelium was present in a part of the cyst wall, suggesting that the cystic shape was caused by obstruction of the pancreatic duct due to enlargement of the tumor originating within the pancreatic duct. In addition, the cystic lesion was accompanied by repeated hemorrhage and a pseudoaneurysm, which made differentiation from a pancreatic pseudocyst challenging.

Hemorrhage from a pseudoaneurysm in a pancreatic pseudocyst is indicated by clinical findings such as upper gastrointestinal bleeding or hemosuccus pancreaticus.^12)^ TAE is a useful approach for controlling bleeding and avoiding surgery. In this case, serum lipase levels were occasionally elevated but not consistently high, and no subcutaneous fat necrosis or polyarthritis was observed. The formation of the aneurysm may have been caused by inflammation of the tumor itself or by inflammation caused by obstruction of the pancreatic duct due to tumor growth. Trypsin levels were not measured during the initial surgery, but at the time of recurrence, they showed a tendency to increase along with tumor growth, suggesting that pancreatic enzymes may have contributed to the formation of the pseudoaneurysm.

The prognosis for acinar cell carcinoma is poor, with a 9%–10% 5-year survival rate in patients who do not undergo resection. The 5-year survival rate for patients who undergo resection is 36%–42%.^13,14)^ Consequently, surgery is the 1st-choice treatment for patients with acinar cell carcinoma without distant metastasis. Compared to conventional pancreatic ductal carcinoma, long-term survival can be expected if resection can be performed. Chemotherapy is generally used for recurrent or metastatic pancreatic acinar cell carcinoma, although no standard regimen has been established. Recent case reports have described treatment with gemcitabine plus nab-paclitaxel, FOLFIRINOX (leucovorin, 5-fluorouracil, irinotecan, and oxaliplatin), or FOLFOX (leucovorin, 5-fluorouracil, and oxaliplatin).^15)^ Meanwhile, surgical resection has been reported as effective in selected cases of localized recurrence or liver metastasis.^16,17)^

In this case, a pseudocyst was initially suspected. However, surgical resection was undertaken due to recurrent bleeding and inadequate control with interventional radiology alone, Histopathological examination of the resected specimen led to a diagnosis of pancreatic acinar cell carcinoma. At recurrence, the possibility of peritoneal dissemination was considered; however, no new lesions were detected after chemotherapy, and the recurrent tumor remained localized, allowing for repeat surgical resection. The patient has remained recurrence-free for an extended period following re-resection, suggesting the effectiveness of surgical intervention in this setting.

## CONCLUSIONS

Acinar cell carcinoma rarely presents with a cystic structure and is rarely associated with a pseudoaneurysm, both of which can complicate differentiation from a pancreatic pseudocyst. This highlights the importance of careful differential diagnosis and the need for further diagnostic evaluation to ensure appropriate treatment.
